# Extract of Calyces from *Physalis peruviana* Reduces Insulin Resistance and Oxidative Stress in Streptozotocin-Induced Diabetic Mice

**DOI:** 10.3390/pharmaceutics14122758

**Published:** 2022-12-09

**Authors:** Ivonne Helena Valderrama, Sandra Milena Echeverry, Diana Patricia Rey, Ingrid Andrea Rodríguez, Fátima Regina Mena Barreto Silva, Geison M. Costa, Luis Fernando Ospina-Giraldo, Diana Marcela Aragón

**Affiliations:** 1 Departamento de Farmacia, Universidad Nacional de Colombia, Av. Carrera 30 # 45-03 Edif. 450, Bogotá 111321, Colombia; 2 Departamento de Bioquímica, Centro de Ciências Biológicas, Universidade Federal de Santa Catarina (UFSC), Campus Universitário, Bairro Trindade, Cx. Postal 5069, Florianópolis 88040, SC, Brazil; 3 Departamento de Química, Pontificia Universidad Javeriana, Av. Carrera 7 # 40–62, Bogotá 110231, Colombia

**Keywords:** flavonoid, rutin, antioxidant, lipidosis, hyperglycemia, diabetes

## Abstract

Diabetes mellitus is a metabolic disorder mainly characterized by obesity, hyperglycemia, altered lipid profile, oxidative stress, and vascular compromise. *Physalis peruviana* is a plant used in traditional Colombian medicine for its known activities of glucose regulation. This study aimed to evaluate the anti-diabetic activity of the butanol fraction from an extract of *Physalis peruviana* calyces in two doses (50 mg/kg and 100 mg/kg) in induced type 2 diabetic mice. Blood glucose levels were evaluated once a week, demonstrating that a dose of 100 mg/kg resulted in greater regulation of blood glucose levels in mice throughout the experiment. The same overall result was found for the oral glucose tolerance test (OGTT) and the homeostatic model assessment for insulin resistance (HOMA- IR). The lipid profile exhibited improvement compared to the non-treated group, a dose of 100 mg/kg having greater protection against oxidative stress (catalase, superoxide dismutase, and malondialdehyde levels). Histopathological findings in several tissues showed structure preservation in most of the animals treated. The butanol fraction from *Physalis peruviana* at 100 mg/kg showed beneficial results in improving hyperglycemia, lipidemia, and oxidative stress status, and can therefore be considered a beneficial coadjuvant in the therapy of diabetes mellitus.

## 1. Introduction

*Physalis peruviana* (Solanaceae) grows at high altitudes in countries of the South American Andes; in Colombia, it grows at 2200 m above sea level (m.a.s.l) [1]. Colombia produces a significant amount of fruits annually; around 15,000 tons/hectare of fruits were collected in 2019 [2]. *P. peruviana* is known as Gold Cape Gooseberry, Poha berry, and Peruvian groundcherry. In Latin counties it is called *uvilla*, *aguaymanto*, *topo*, *uchuva* and *capulí* [3]. Calyces cover the fruits and protect them from different exogen factors, such as birds, insects, inclement changes in weather, and pathogens [4]. In traditional medicine and ethnomedicine around the world, *Physalis peruviana* is used to treat different disorders: the leaves and stems to treat cancer, fungal infections, and malaria; the fruits to treat diabetes, cataracts and conjunctivitis, and typhoid fever, and the aerial parts to treat diabetes, cholic in children, malaria, pneumonia, and postpartum pain [5].

Several reports have confirmed the antihyperglycemic and antioxidant effects of several parts of the plant. Authors have previously shown the anti-diabetic activity from fruits, regulating blood glucose levels and attenuating oxidative stress [6]. Studies have also shown the inhibition of some intestinal carbohydrases using an extract from *P. peruviana* [7]. In 2006, different extracts obtained from the leaves of this plant were studied; scavenging activity on superoxide anion and xanthine oxidase inhibition was studied, as well as its anti-inflammatory activities. All these activities were attributable to the flavonoid content [8]. The calyces have demonstrated antioxidant and anti-inflammatory activities of this harvest by-product [6,9,10] and it has been clearly demonstrated that in extract of *P. peruviana* calyces, rutin (Quercetin 3-*O*-rutinoside) is the main flavonoid [11]. Beneficial effects, such as antioxidant effects and blood glucose level regulation, have been attributed to this flavonoid [12]. This study aimed to determine the effect of the butanol fraction from an extract from calyces of *P. peruviana,* to determine the content of the most active extract obtained in the order of related in vitro and in vivo experiments, and to determine its anti-diabetic potential. A diabetic mice model, induced by a high-fat diet and administration of streptozotocin (STZ) in repeated low doses, was chosen because it is the model most commonly used in the physiopathological study of diabetes mellitus, and because it enables outcomes to be determined in a dose-dependent manner [13]. 

## 2. Materials and Methods

### 2.1. Materials

Ethanol 99.5% (PanReac AppliChem, Darmstadt, DEU) was used to prepare the *P. peruviana* extract. Formic acid reagent grade (Merck, Rahway, NJ, USA), acetonitrile chromatography grade (Merck), and ultrapure water (Milli-Q system Millipore^®^, Rahway, NJ, USA) were used for the liquid chromatographic analysis. 

Standar LabDiet^®^ (5001, Richmond, IN, USA), DIO Rodent Purified Diet with 45% Energy from Fat-Red (58V8), Tween^®^ 80, carboxymethyl cellulose-CMC (419273), glucose (G7021), streptozotocin (572201), a catalase assay kit (CAT100), a lipid peroxidation (MDA) assay kit (MAK085), and a SOD assay kit (19160) were purchased from Sigma-Aldrich^®^ (St. Louis, MI, USA). An ELISA monobind insulin kit (2425-300A) was used to determine serum insulin levels. A triglyceride MR kit, cholesterol MR kit (1118005), LDL-cholesterol kit (1133105), and HDL-cholesterol kit (1133010) were used to measure lipid markers. These were purchased from Linear Chemicals SLU (Montgat, ESP). Pentobarbital sodium was purchased from Invet (Bogotá, COL).

### 2.2. Plant Material

The calyces of *Physalis peruviana* were collected in Granada-Cundinamarca (Colombia), dried at 40 °C, ground, and sifted [14]. A voucher specimen was stored in the Herbarium of the National University of Colombia (COL 512200). The plant material was identified by taxonomist Parra C.

#### Preparation of the Extract and Fractions

Hydroethanolic extract of *P. peruviana* (HEPP) was prepared according to the standardized methodology of the same research group [14]. For every 10 g of vegetal material, 150 mL of solvent was added (drug:solvent ratio 1:15) using 70% ethanol. The extraction was performed by percolation after 72 h, collecting the percolated product every 24 h. Finally, the ethanol of the extracts was evaporated under reduced pressure, and the remaining water was eliminated by freeze-drying for 24 h (yield: 9.56%). HEPP was submitted to fractionation using, successively, dichloromethane, ethyl acetate, butanol, and water [15]. The dichloromethane fraction was not used due to its low yield (0.9%). The aqueous fraction (WFPP) was frozen and then lyophilized (yield: 10.84%). Ethyl acetate (EFPP) (yield: 39.27%) and BFPP (yield: 12.34%) were evaporated to dryness in a vacuum centrifuge. The fractions were stored at 4 ± 2 °C until further analysis. Preliminary screening studies were conducted to select the most promising fraction.

### 2.3. Dereplication Analysis

From the butanol fraction obtained from the hydroethanolic extract of calyces of *P. peruviana,* separation was performed using an RP-18 column (Phenomenex Luna Omega 1.6 µ, 2.1 × 150 mm, 100 A) and, as mobile phases, 0.2% formic acid aqueous solution (A) and acetonitrile: methanol (80:20) acidified with 0.2% formic acid (B). Gradient elution was performed as follows: 0–5 min 5% B isocratic conditions; 5–28 min 5–70% B; 28–33 min 70–98% B; 33–43 min 98% B; 43–43.1 min 98-5% B; and 43.1–45 min 5% isocratic conditions. The samples were dissolved in methanol. The flow rate of the mobile phase was 0.25 mL/min at 40 °C. A Thermo Scientific Ultimate 3000 HPLC coupled with A maXis HD QTOF mass spectrometer (Bruker, Germany) was used. Chromatography parameters were determined using positive and negative ionization modes (100–1500 Da). A capillary voltage from +2700 V to −2500 V), a nebulizer pressure of 0.4 Bar, a dry gas flow rate of 4 L/min and a dry gas temperature of 200 °C were used as the chromatographic conditions. The results were analyzed using the software program Bruker Compass Data Analysis 4.2.

Complementary analysis was performed to corroborate the presence of molecules previously reported in the literature. This was done using a Nexera UHPLC coupled to a mass spectrometer with ESI ionization and triple quadrupole (TQ) (LCMS 9030 Shimadzu Scientific Instruments^®^, Columbia, MD, USA). Spectrophotometric conditions were the same as those mentioned above. The mass spectrometer was operated in positive and negative ionization modes, and data were obtained at between 100 and 2000 atomic mass. Capillarity conditions were a voltage +3 kV, a sample cone voltage of 33 kV, a dry temperature of 250 °C, nitrogen nebulizer gas at 350 L/h, argon collision gas at 50 L/h, and a collision energy of 2.5 eV. The data were processed using the software program Lab Solutions.

### 2.4. Animals

For the screening OGTT assay, normoglycemic female Wistar rats 7–10 weeks old (180–200 g) were used to evaluate the hypoglycemic activity of the HEPP and its fractions. During acclimatization, water and food were available ad libitum [16].

The diabetic model was performed using CD-1 female mice at 16 weeks old, weighing about 20–25 g. From the beginning until the end of the experiment, animals received a high-fat diet (Test Diet^®^ DIO Rodent Purified Diet 45% Energy from Fat-Red 58V8) and water ad libitum. Body weight and blood glucose levels were checked once a week. 

All animals were obtained from the Pharmacy Department of the National University of Colombia. The mice were housed in optimal humidity and temperature conditions (22 °C ± 2 °C) with 12-h light/dark cycles. The study was approved by the local Research Ethics Committee (Act 08 of 16 April 2020, Faculty of Science).

### 2.5. Diabetic Chronic Model Induction and Treatments

In the first part of the experiment, all animals received a daily high-fat diet for eight weeks (protein 20.8%; fat 23.6%; fiber 5.8%; carbohydrates 41.2%; metabolizable energy (kcal/g)^2^ 4.6). At the end of this period, streptozotocin was administered via intraperitoneal (i.p.) injection (STZ-40 mg/kg) (Sigma Chemical Company, St. Louis, MO, USA) twice, with an interval of five days between each injection [17]. STZ was prepared in citrate buffer (pH 4.5) and applied once, attempered and stabilized [18]. Immediately before and during the first 24 h, animals were provided with a 5% glucose solution overnight to avoid drug-induced hypoglycemia. Three days after the last administration of STZ, blood glucose levels (BGL) were measured using an Accu-Chek Performa^®^ device; blood samples were obtained from a small incision in the caudal vein. For the classification of diabetic animals, mice with BGL above 150 mg/dL were considered suitable for the next phase of the experiment. The diabetic mice were randomly distributed into four groups (n = 6). All treatments were administered for 21 days through an orogastric tube: vehicle CMC 0.5%-Tween 80 0.5%; metformin 250 mg/kg; BFPP 50 mg/kg, and BFPP 100 mg/kg. Additionally, a normoglycemic group was fed on a regular diet (protein 24.1%; fat 6.4%; fiber 5.3%; carbohydrates 25.2%;metabolizable energy (kcal/g)^2^: 3.35). This group was assigned as additional control, and received saline solution as treatment. 

At the end of the experiment, all the animals were sacrificed by cervical decapitation after i.p. injection of pentobarbital sodium (60 mg/kg) [19]. Blood samples were obtained immediately and centrifuged (3500 rpm for 10 min) to collect the serum supernatant. The organs were removed, washed with saline buffer, conserved at –80 °C for subsequent assays, and then placed in formalin 10% for histopathological analysis.

#### 2.5.1. Oral Glucose Tolerance Test (OGTT)

After 4 h of fasting, animals received each treatment 30 min before an oral glucose overload (2000 mg/kg). BGL was measured before and after glucose administration and every 30 min (30, 60, and 90 min) [20]. 

#### 2.5.2. HOMA-IR

An ELISA kit was used to measure insulin levels. Insulin resistance (IR) was determined according to the homeostasis model assessment index, which was calculated based on fasting serum insulin and blood glucose levels at the end of the experiment. HOMA-IR was determined by the following formula [21]:

(1) $$\mathrm{HOMA}-\mathrm{IR}=\frac{\left(\mathrm{plasma}\;\mathrm{insulin}\;\mathrm{level}\;\left[\mu U/\mathrm{mL}\right]\times \;\mathrm{fasting}\;\mathrm{plasma}\;\mathrm{glucose}\;\left[\mathrm{mg}/\mathrm{dL}\right]\right)}{405}$$

### 2.6. Biochemical Parameters

#### 2.6.1. Oxidative Stress Parameters

Commercial kits were used to evaluate different parameters of oxidative status in the tissues of the euthanized animals. Malondialdehyde (MDA), catalase (CAT), and superoxide dismutase (SOD) in liver, pancreas, and kidney homogenates were determined.

#### 2.6.2. Serum Lipid Profile

Total cholesterol (TC), triglycerides (TG), low-density lipoprotein (LDL), and high-density lipoprotein (HDL) levels were measured in the diagnostic kit described previously. 

### 2.7. Histological Analysis

Postmortem, the pancreas was removed, weighed, and placed in a 10% phosphate-buffered formaldehyde solution at a ratio of 1:20. The samples were placed and cut into thin slices of 5 µm. Finally, the sections were stained with hematoxylin & eosin.

## 3. Statistical Analysis

The software program GraphPad Prism^®^ (version 6, San Diego, CA, USA) was used for statistical analysis. The results are expressed as mean ± SEM. ANOVA analysis of variance (ANOVA) and Bonferroni or Dunnett’s test for multiple comparisons. *p* ≤ 0.05 was considered statistically significant.

## 4. Results

### 4.1. Chemical Characterization

**Figure 1:**
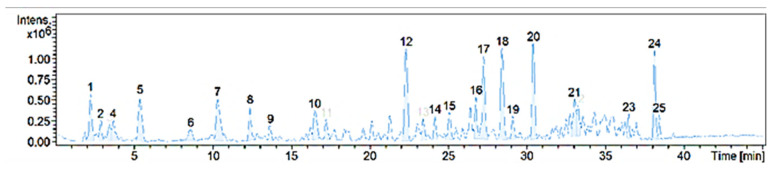
Total ion chromatogram of BFPP in the positive ion mode.

The chemical composition of BFPP confirmed the content of the flavonoid quercetin 3-*O*-rutinoside (#10). Figure 1 shows the compounds detected, followed by a list of the possible chemical identification using ACD/Labs C NMR Predictor software.

As can be seen in Table 1, Table 2 and Table 3, several compounds constitute the matrix of the fraction. Several of these compounds have been studied for their protective activities against cellular damage, as well as their regulatory activity on some diabetes disease markers. Phenolic acid derivates of cinnamic acid (caffeic acid and coumaric acid) obtained from red wine were shown to provide a protective effect on human serum and low-density oxidation [22]. The presence of derivates from hydroxycinnamic acids, such as chlorogenic acid, feruloylquinic acid, and neochlorogenic acid, reduced proinflammatory markers and improved the anti-inflammatory response and parameters over blood oxidative stress in the presence of fractions from *Herniaria polygama* [23,24]. 

### 4.2. Diabetic Model

#### 4.2.1. Effect of Butanol Fraction from *P. peruviana* on Blood Glucose Levels

**Figure 2:**
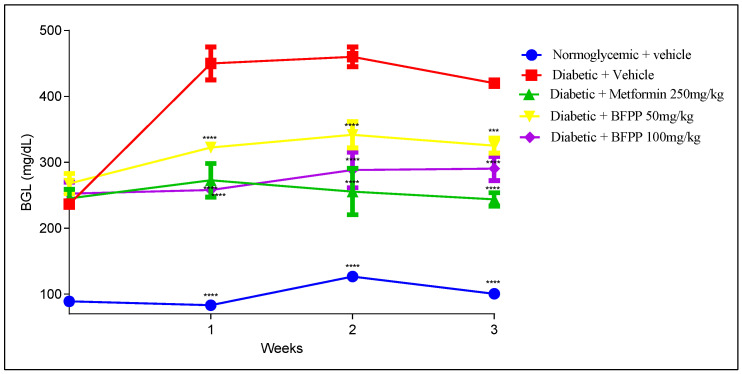
Blood glucose levels (BGL) of animals treated for 21 days. Normoglycemic (blue), vehicle (red), metformin 250 mg/kg (green), BFPP 50 mg/Kg (yellow), BFPP 100 mg/Kg (purple). The data are expressed as mean ± SEM, n = 6 animals per group. Two-way ANOVA post-test Bonferroni; *** *p* < 0.001 and **** *p* < 0.0001 compared with the vehicle group.

The hypoglycemic activity from BFPP was evaluated following BGL over 3 weeks. As shown in Figure 2, the diabetic group not treated (vehicle) reached BGL of up to 400 mg/dL, remaining above that range over time. All the diabetic animals started the experiment with BGL up to 200 mg/dL (fasting BGL from 150 mg/dL were considered diabetic [18]); however, as the graph shows, the group treated with metformin maintained the lowest levels of BGL during treatment, with statistical differences between the vehicle group. Similar behavior from BFPP 100 mg/kg was also observed. BFPP of 50 mg/kg also demonstrated significant differences from the vehicle, reaching BLG of up to 300 mg/kg. Although BGL tended to increase over time when compared to the vehicle group, an anti-hyperglycemic effect was evidenced; this outcome is attributed to content of the flavonoid rutin as the major flavonoid [20]. Previous reports assessed the activity of withanolide, 4-OH-withanolide, and perulactone as anti-diabetic agents present in the ethanolic extract of *P. peruviana,* showing mild action on the insulin tyrosine kinase receptor. Manzano et al., attributed the inhibitory activity on SGLT 1 channels to quercetin-3-*O*-glucoside, quercetin-4-*O*-glucoside. The inhibitory effect of quercetin on GLUT 2 in Caco-2 cells has also been demonstrated [28]. These mechanisms prevent hyperglycemia and support the anti-diabetic treatment target.

Once ingested, most of the rutin flavonoid undergoes a deglycosylation in intestinal tissue mediated by the catalytic effect of α-rhamnosidase and β-glucosidase from intestinal microbiota. The flavonoid quercetin remains after of conjugation with sulfate or glucuronic acid in intestinal villi. It was shown that quercetin-3-O-sulfate (Q3OS) and quercetin-3-O-glucuronide (Q3OG) are the principal molecules found in plasma when rutin is administrated orally. Quercetin molecules can flow in the blood circulation through P-glycoprotein membrane efflux [29]. Previous studies of our research group demonstrated that the matrix from the extract of calyces from *P. peruviana,* improved rutin clearance and volume of distribution. Additionally, the absorption and oral bioavailability of the conjugates was evidenced [30].

**Figure 3:**
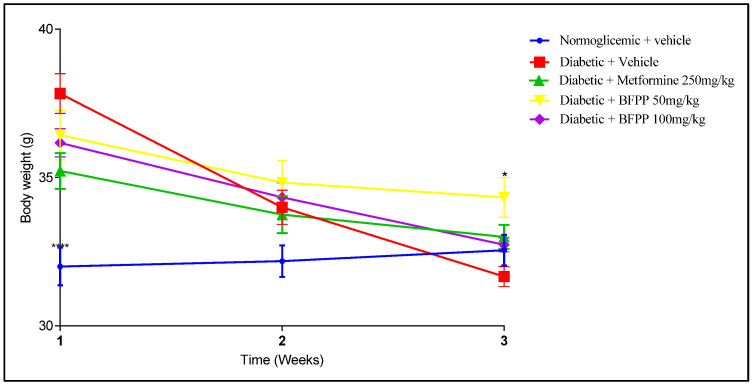
Effects of BFPP on body weight of mice with induced diabetes. Normoglycemic (blue), vehicle (red), metformin 250 mg/kg (green), BFPP 50 mg/kg (yellow), BFPP 100 mg/kg (purple). The data are expressed as mean ± SEM. Two-way ANOVA post-test Bonferroni. * *p* ≤ 0.05 and **** *p* ≤ 0.0001 compared with the vehicle group.

As can be seen in Figure 3, weight loss became apparent from the start of treatments until after the last administration of STZ. However, the animals remained overweight when compared with the control group. This finding is consistent with those of other studies, without significant statistical differences between treatments [17]. Only the NO-treated group (vehicle) continued to lose weight, which is consistent with the progress of the disease and with the injury caused by STZ, including damage to the pancreatic islets, massive β-cell damage, sustained inflammation, damage to other organs, and insufficient insulin production resulting in hyperglycemia [18]. Regarding Metformin and BFPP, although they caused weight loss, this weight loss was not progressive, and the mice did not regain weight but stayed at the same weight. Catabolic processes were improved.

#### 4.2.2. Oral Glucose Tolerance Test (OGTT)

**Figure 4:**
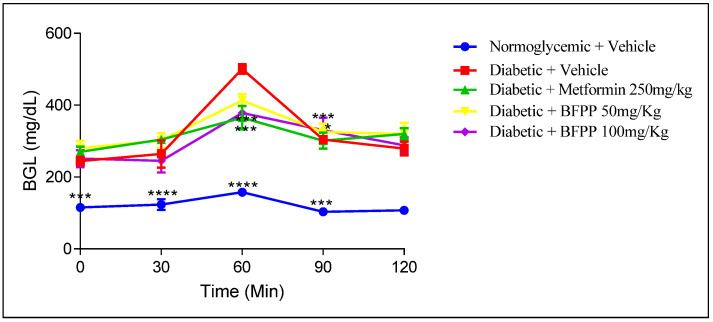
Hypoglycemic activity of BFPP on diabetic mice. Normoglycemic (blue), vehicle (red), metformin 250 mg/kg (green), BFPP 50 mg/Kg (yellow), BFPP 100 mg/Kg (purple). The data are expressed as mean ± SEM, n = 6 animals per group. Two-way ANOVA post-test Bonferroni; * *p* ≤ 0.05; *** *p* < 0.001 and **** *p* ≤ 0.0001 compared with the vehicle group.

At the end of the treatments, an OGTT was carried out to determine the capability of the animals to regulate postprandial glucose levels. Figure 4 shows similar levels of BGL between the metformin group and BFPP 100 mg/kg at 60 min, with statistical differences when compared with the vehicle group. A BFPP of 100 mg/kg represented the lowest BGL compared to the other treatment at 60 min.

Several studies support these results in relation to several means of regulation. One such means is the inhibitory activity on different intestinal carbohydrases from the *P. peruviana* extract, as has been reported in previous studies [7,31]. Additionally, effects on α- amylase have been attributed to the presence of O-glycosyl flavonoids, as identified in BPFF [32]. Other mechanisms include muscular glucose uptake and insulin secretagogue, which are attributed to the flavonoid rutin content [33]. Additional investigations cite polyphenols as being responsible for inducing GLP-1 secretion by activation of GPCRs (G-protein coupled receptors) to regulate intracellular signaling and modulate its production [34]. The n-BuOH and aqueous residual fractions of *M.* × *paradisiaca* leaves were shown to improve glucose homeostasis OGTT in transitory hyperglycemic rats; the authors identified the presence of rutin as its major compound [33].

#### 4.2.3. Homa-IR

Homeostasis model assessment-estimated insulin resistance (HOMA-IR) is a mathematical model that includes interactions between fasting plasma insulin and fasting plasma glucose concentrations. The model has been widely used to estimate insulin resistance in preclinical research, such as epidemiological studies [35,36].

According to the results shown in Table 4, after 21 days of treatment, fasting glucose, insulin, and the HOMA index decreased in mice that received metformin, as was expected, and in agreement with what was previously reported [37]. Furthermore, 50 mg/kg of BFPP decreased fasting glucose by 27.7%, fasting insulin by 57.9%, and HOMA-IR by 70%. Meanwhile, treatment with 100 mg/kg of BFPP reduced fasting glucose by 35.4%, fasting insulin by 58.8%, and HOMA-IR by 73.2%. Notably, the higher dose of the butanol fraction showed more improvement in the three parameters measured than the dose of 50 mg/kg, which may be explained by the higher proportion of flavonoids and phenolic acids present. Other extracts for which the presence of rutin has been reported, and which have shown to improve the HOMA-IR index in a high-fat diet with low-dose streptozotocin-induced type 2 diabetic rats are *Capparis spinosa* fruit extract, also reported as containing kaempferol-3-rutinoside [38], and *Morus alba* ethanol leaf extract, reported as containing chlorogenic acid [39]. It is remarkable that rutin has previously been reported to diminish glycemia by enhancing insulin secretion and stimulating calcium uptake in rat pancreatic islets [36]. The decrease in insulin resistance can be attributed to this flavonoid. Finally, it should be noted that 25 mg/kg or 50 mg/kg of rutin has been shown to decrease the HOMA-IR index in twenty-month-old rats [40]. We can conclude that the decrease in insulin resistance caused by BFPP can be attributed to the presence of rutin.

#### 4.2.4. Effect of BFPP on Lipid Profile

**Figure 5:**
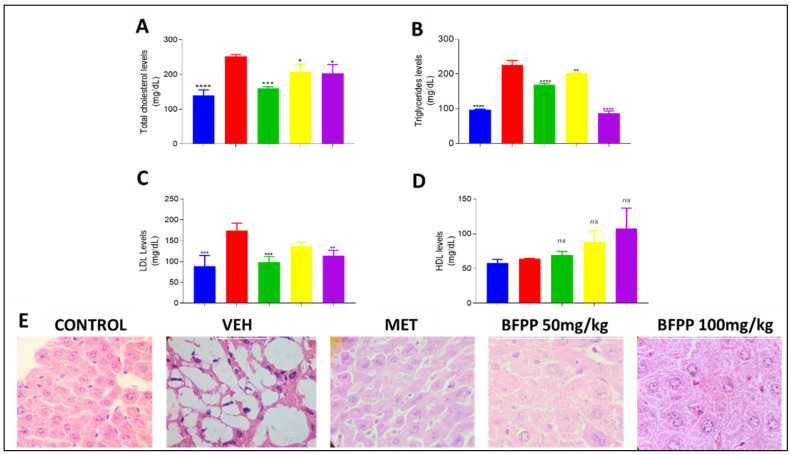
Effect of BFPP on lipid profile parameters. Normoglycemic (blue), vehicle (red), metformin 250 mg/kg (green), BPFF 50 mg/kg (yellow), BFPP 100 mg/kg (purple). The data are expressed as mean ± SEM n = 6 animals per group. One-way ANOVA post-test Dunnet; *ns*: not significant; * *p* ≤ 0.05; ** *p* ≤ 0.01, *** *p* < 0.001 and **** *p* ≤ 0.0001 with respect to the vehicle group. (**A**) Total cholesterol levels; (**B**) triglyceride levels; (**C**) LDL levels; (**D**) HDL levels; (**E**) Liver histology (CONTROL: Normoglycemic group; VEH: diabetic group which received only vehicle; MET: Metformin 250 mg/kg; BFPP 50 mg/kg and BFPP 100 mg/kg.

Lipid profile markers showed a protective effect of BFPP at both doses (Figure 5); however, the group which received 100 mg/kg showed a more marked attenuating effect on TG, reaching a lower level than even the group treated with metformin. LDL levels were similar among the same groups. This attenuation in the other groups suggests the beneficial effect of BFPP on lipid profile. HDL showed no differences compared to the vehicle group.

Lipidemic disorders have been considered responsible for human mortality in the last decade, and studies on the effects of polyphenol compounds have demonstrated beneficial improvements on hyperlipidemic status [41]. For example, previous studies have demonstrated the effects of ferulic acid in decreasing TG and cholesterol levels [42]. Similar findings were reported when studying the antilipidemic effects of zedoary (*Curcuma zedoaria Roscoe*) herbal tea in humans. These effects were related to the phenols contained among its constituents [43]. It also improved the lipid profile in young rats with induced insulin resistance and then supplemented with peel flour of *Passiflora edulis* Var. Flavicarpa. These effects were also attributable to a high amount of flavonoids (quercetin-3-*O*-glucoside) and caffeic acid [44]. A derivate of rutin was demonstrated to attenuate cholesterol levels in rats induced to diabetes by alloxan application [45]. *P. peruviana* pomace also decreased the total cholesterol between 23% and 35% in rats and increased HDL cholesterol levels [46]. Similar results were found when STZ diabetic rats were treated with pomace and juice from *P. peruviana,* corroborating the beneficial effect on lipid profile parameters. These effects were related to the presence of flavonoids and vitamin C [47]. Figure 5E shows the difference between the livers of animals that received the different treatments. The normal group shows normal liver architecture, with clearly-defined hepatic cords, while the vehicle group presents hypertrophic adipocytes, a finding compatible with marked steatosis, which would be expected based on the triglyceride levels achieved. The metformin group shows a small peripheral white area of cytoplasm corresponding to lipid deposits, making this more similar to the BFPP 50 mg/kg group. In fact, BFPP 100 mg/kg showed the greatest preservation of liver structure than the vehicle group, with no evidence of steatosis and a better response with respect to triglyceride levels. In general, BFPP treatments showed a limited presence of binucleated cells as a typical sign of regeneration, demonstrating the protective response in liver tissue. These results are in agreement with previous reports that attribute dyslipidemia attenuation by the flavonoid content in an extract from *Sophora alopecuroides* L. in HFD and STZ-induced diabetic mice [17]. Another study demonstrated the effect of the flavonoid rutin on lipid accumulation in older rats, decreasing liver injury and related complications, such as insulin resistance and chronic systemic inflammation [40].

#### 4.2.5. Effect of BFPP on Oxidative Stress Markers

ROS production is essential for the development of tissue damage in diabetes mellitus [48]. This study evaluated some parameters of oxidative stress in diabetic mice in association with other findings observed during the treatments and post-mortem. ROS are a critical cause of beta cell deterioration and cellular damage produced after STZ treatment, as determined by DNA alkylation and DNA fragmentation. The author hypothesizes as to the diabetogenic capability of streptozotocin such as nitric oxide (NO) speeding up pancreatic cell damage [49]. 

**Figure 6:**
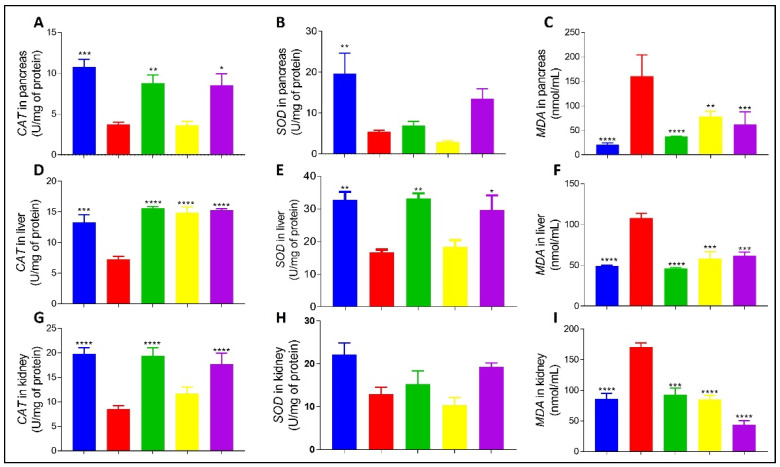
Effect of BFPP on oxidative stress markers. Normoglycemic (blue), vehicle (red), metformin 250 mg/kg (green), BFFF 50 mg/kg (yellow), BFPP 100 mg/kg (purple); (**A**) pancreas CAT; (**B**) pancreas SOD; (**C**) pancreas MDA levels; (**D**) liver CAT; (**E**) liver SOD; (**F**) liver MDA levels; (**G**) kidney CAT; (**H**) kidney SOD; (**I**) kidney MDA levels. The data are expressed as mean ± SEM, n = 6 animals per group. One-way ANOVA post-test Dunnet; * *p* ≤ 0.05; ** *p* ≤ 0.01, *** *p* < 0.001 and **** *p* ≤ 0.0001 compared with the vehicle group.

As can be observed in Figure 6A,B, enzyme activities were maintained in the pancreas, with CAT being active in the group treated with metformin and showing very similar behavior to BFPP 100 mg/kg. These results were statistically significant compared to the vehicle group. SOD activity was greater in the animals that received BFPP 100 mg/kg, and even greater than in the metformin group, suggesting the protective activity of the fraction to this dose. An important protective activity against lipid peroxidation (MDA) is shown in Figure 6C, where a protective response can be observed concerning the negative control group with significant differences compare with untreated group. BFPP significantly inhibited MDA production in a dose-dependent manner. These findings are consistent with previous research that reported the protective effect against lipid peroxidation and increased SOD and CAT levels in the pancreas of STZ-diabetic rats treated with *P. peruviana* juice and pomace [6,47].

As shown in Figure 6D, the livers from all groups had high CAT activity compared with the negative control, with similar levels of enzymatic activity. Figure 6E shows SOD activity in groups treated with BFPP in a dose-dependent manner. The BFPP 100 mg/kg group showed similar behavior to the group treated with metformin. MDA levels were statistically different in all groups compared with the vehicle group. Both doses of BFPP showed protective enzyme activity from the butanol fraction of *P. peruviana* on this organ (Figure 6F). Unlike other organs, the liver is much better equipped to prevent the formation of hydrogen peroxide by CAT endogen levels, compared to the pancreas, for example [49]. Previous findings corroborate the hepatoprotective effect of *P. peruviana* calyces by neutralization of ROS by CAT and SOD enzymes in a liver inflammation model induced by CCl_4_ [50]. Marked differences between the residual activity of SOD in the pancreas and kidney, as compared to the liver, can be explained by the lower intrinsic capability of these organs to express this antioxidant enzyme gene, with the pancreas expressing it to a lesser extent [51].

As shown in Figure 6G, CAT in the kidney was active in all the treated groups, but in BFPP, the best outcome was observable at a dose of 100 mg/kg. Figure 6H shows the activity of SOD but without statistical differences in relation to the vehicle. MDA levels in Figure 6G show the protective activity of all treatments compared to the untreated group. The importance of the antioxidant effect on the kidney in a diabetic individual is the risk of gradual structural and functional loss, which was countered in diabetic rats induced by streptozotocin and treated with *P. peruviana* juice [6,47]. Other studies have reported that these results can be associated, in part, with the presence of rutin in BFPP, which corroborates the finding that its flavonoid improves oxidative stress [40].

#### 4.2.6. Histopathological Analysis

**Figure 7:**
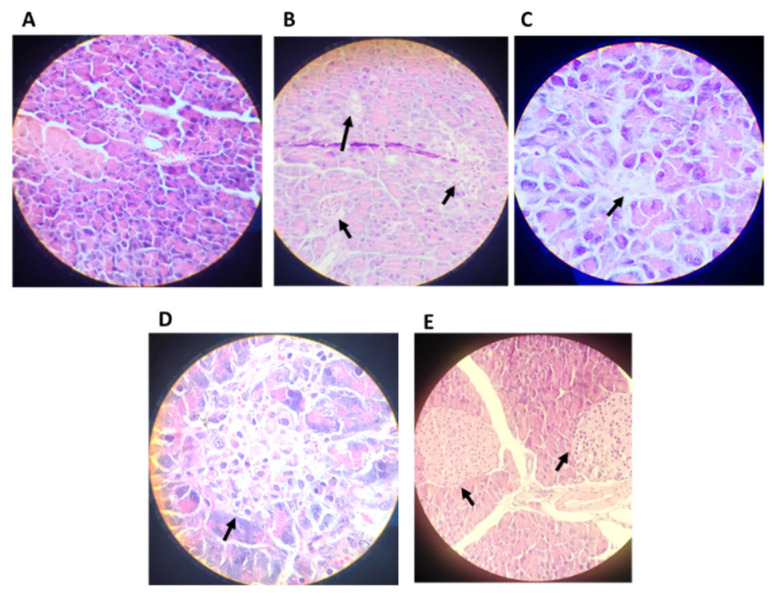
Effect of the butanol fraction from calyces of *P. peruviana* on pancreatic islets at 50 mg/kg and 100 mg/kg. (**A**) Untreated diabetic group (vehicle); (**B**) normal control group; (**C**) diabetic group treated with metformin; (**D**) diabetic group treated with BFPP 50 mg/kg; (**E**) diabetic group treated with BFPP 100 mg/kg. Micrographs were stained with H&E, with a magnification of 40× and 100×. Arrows shows the presence of Langerhans islets.

As shown in Figure 7, animals treated with BFPP maintained the presence of granules, especially FBPP 100 mg/kg (Figure 7E). The architecture of the islets was preserved without hypertrophy. At the dosage of 50 mg/kg (Figure 7D), the glandular tissue was protected, but not enough to preserve the entire islet compartment to avoid diffusion of the islets. Comparing the diabetic untreated group (Figure 7A) with the other groups, we saw an evident absence of glandular tissue, which is usually a consequence of the initial tissue inflammation and hypertrophy. The metformin group (Figure 7C) demonstrated the presence of Langerhans islets, without enlargement, and a preserved architecture similar to that of the control group (Figure 7B), which is consistent with the respective responses on BGL. However, BFPP 100 mg/kg showed the best parenchymal and granular protection and BGL attenuation.

**Figure 8:**
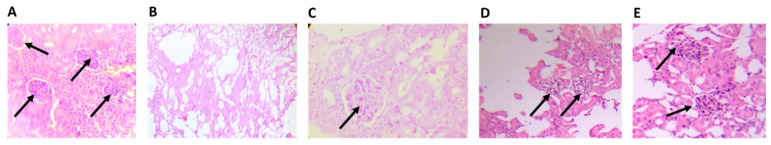
Effect of the butanol fraction from calyces of *P. peruviana* on kidney tissue at 50 mg/kg and 100 mg/kg. (**A**) Untreated diabetic group (vehicle); (**B**) normal control group; (**C**) diabetic group treated with metformin 250 mg/kg; (**D**) diabetic group treated with BFPP 50 mg/kg; (**E**) diabetic group treated with BFPP 100 mg/kg. Micrographs were stained with H&E, with a magnification of 40× and 100×. Arrows show the presence of renal glomeruli.

As seen in Figure 8A, the control group showed a normal tissue architecture with the presence of glomeruli, capillary loops and Bowman space and capsule. Sections from the vehicle group showed a total absence of the glomeruli and tubular disruptions (Figure 8B). The groups treated with BFPP fraction showed the presence of glomeruli, and at a higher dose (Figure 8E), greater preservation of the architecture. Some hemorrhagic foci were evidenced at the lower dose of BFPP (Figure 8D). The metformin group showed a normal appearance with the presence of glomeruli and preserved tissue architecture. A previous study, which tested ethanolic extract from fruits of *P. peruviana* and its ethyl acetate subfraction, showed the beneficial effects on nephropathy in rats with streptozotocin-induced diabetes. Among the identified compounds, several of them are also present in BFPP, such as kaempferol, quercetin and *O*-caffeoylquinic acid derivates. This mode of action has been associated with apoptosis inhibition and the promotion of autophagy. The authors suggest that *P. peruviana* offers a reno-protective effect on diabetic disorders [52]. To further investigate the effect of BFPP on kidney tissue, further studies are recommended to analyze the markers of kidney disease. Previous reports have mentioned the beneficial and protective effect of the polyphenols contained in an extract of *Hibiscus sab dariffa* calyx, as a coadjuvant in the treatment of type 2 diabetic rats. Caffeoylquinic acid derivates, caffeic acid, feruloyl derivative, quercetin derivative and, kaempferol-3-glucoside were some of the molecules identified [53]. 

## 5. Conclusions

Natural products with antioxidant activities are used as adjuvants to treat several diseases. Several parts of *P. peruviana* have been studied for their antioxidant activity and anti-diabetic effects [54], and they have been used in traditional medicine to treat several diseases related to oxidative stress. In this study, the butanol fraction from *P. peruviana* showed remarkable antioxidant activity in several tissues, as well as an antihyperlipemic effect and glucose regulation. As observed, in addition to the content of the rutin flavonoid (major compound), BFPP is rich in several molecules that can act synergistically, with potentially beneficial effects. Caffeic acid derivates present in BFPP have demonstrated antioxidant activity in other studies, and withanolide derivates (withanona) have been related to the antioxidant potential of *Withania somnifera* [22,55,56]. Some amino acids present in BFPP (alanine, leucine, and isoleucine) have been previously reported for their activity on insulin secretion stimulation. This is preliminary information that may open the way for more in-depth study of the anti-diabetic modes action of the butanol fraction extracted from the calyx. Molecules present in BFPP can explain the protective activities of the previously studied oxidative process.

The results of this study suggest that the butanol fraction from *P. peruviana* is a natural source of anti-diabetic compounds. *P. peruviana* could be considered for use as an adjuvant for the treatment of diabetes mellitus. At the end of the treatment, different lipid and oxidative stress markers and BGL were attenuated in a dose-dependent manner. 

## Figures and Tables

**Table 1:** Compounds identified in BFPP by the dereplication method.

#	Max. *m/z*	RT.	Area	Possible Compound	Possible ID (CAS.)
1	116.0708	2.2	6680138	Amino Acid: L-Proline	147-85-3
2	142.1230	2.9	2810985	Tropane Alkaloids:	
				(1) Tropine	120-29-6
				(2) Physoperuvine	60723-27-5
				(3) Cycloheptanone, 3-(methylamino)	73744-99-7
3	132.1024	3.4	2759332	Amino Acid: L-Isoleucine	61-90-5
4	132.1024	3.7	3675771	Amino Acid: S-Leucine	73-32-5
5	166.0868	5.4	8761530	Amino Acid: Phenylalanine	63-91-2
6	251.1399	8.6	2877906	Cinnamic Acid Derivatives: Cinnamamide, N-(4-aminobutyl)-3,4-dihydroxy-	8CI; 29554-26-5
7	205.0979	10.3	9139461	Amino Acid: L-Tryptophan	73-22-3
8	265.1555	12.4	45393229	Cinnamic Acid Derivates: Cinnamamide, N-(4-aminobutyl)-4-hydroxy-3-methoxy-	7CI,8CI; 501-13-3
9	474.2610	13.7	2022964	N/I	N/I
10	611.1622	16.5	5663280	Flavanoid: Flavone, 2′,3,4′,5,7-pentahydroxy-	8CI; 480-16-0
11	1037.5117	17.2	2652622	Withanolides: Withaperuvin E	92125-38-7
12	1005.5214	22.3	14971186	Withanolides: Withaperuvin M	1353093-20-5
				Phyperunolide A	1198400-48-4
13	233.1023	23.4	3245775	N/I	N/I
14	331.0817	24.1	2913771	Terpene: Dihydroactinidiolide	17092-92-1
15	247.1182	25.1	4087021	N/I	N/I
16	977.5637	26.8	4565672	Withanolides:	
				(1) Withaferin A	5119-48-2
				(2) Withanone	7CI; 27570-38-3
				(3) Withanolide D	30655-48-2
				(4) 27-Hydroxywithanolide B	60124-17-6
17	203.1797	27.2	12844741	Monoterpene: Calamenene	73209-42-4
18	345.0974	28.4	14689013	Retinol	68-26-8
19	316.2853	29.1	3375421	Monoterpene: β-Vetivenene	27840-40-0
20	345.2429	30.4	13726374	N/I	N/I
21	289.2535	33.0	5550203	Biotin	58-85-5
22	289.2533	33.2	3910919	Fatty Acid: Linolenic acid, ethyl ester	1783-84-2
23	273.2581	36.5	2264958	Flavanoid: Flavanone, 4′,5,7-trihydroxy-	8CI; 480-41-1
24	621.3086	38.1	10006030	N/I	N/I
25	621.3088	38.4	2128388	N/I	N/I

N/I: Not identified.

**Table 2:** Additional flavonoids and phytoprostanes present in BFPP detected by LC-MS analysis in negative mode, and corroborated by previous reports in the literature.

Compounds	Rt	[M-H]^−^ *m*/*z*	MS^n^	Hexose (-162)	Aglicone	Author
Quercetin 7-*O-*glucoside 3-*O-*rutinoside	4.17	771 (T)		609	301	[25,26]
Kaempferol 7-*O-*glucoside-3-rutinoside	4.8	755		593		[25,26]
Quercetin 3-*O-*rutinoside	6.8	609 *			301	[10,25,26,27]
Quercetin-3-*O-*glucoside	6.8	463			301	[25,26]
Kaempferol 3-*O-*rutinoside	7.7	593 *			285	[10,25,26,27]
9-Flt-PhytoP	5	327.2 (T)	171.1			[25]

T: Traces. * The greater amount with respect to the other compounds.

**Table 3:** Additional phenolic compounds present in BFPP, detected by LC-MS analysis in negative mode, and corroborated by previous reports in the literature.

Compounds	Rt	[M-H]^−^ *m*/*z*	MS^n^	[AF-H]^−^	[AQ-H]^−^	[AC-H]^−^	[AF-H-18]^−^	[AQ-H-18]^−^	[*p.*CoA-H]^−^	Author
3-*O*-Caffeoylquinic acid	1.9	179 (T)								[26]
3-*p*-Cumaroylquinic acid	2.6	337						173 (T)	166 *	[26]
3-*O*-Feluroylquinic acid	3.2	367		193 *				173 (T)		[26]
5-*O*-Caffeoylquinic acid	3.3	353			191 *	179 (T)				[25,26]
Ferulic acid hexoside	3.5	355	193 *				175			[26]
Ferulic acid hexoside	3.8	355	193 *				175			[26]
Ferulic acid hexoside	4.1	431	385 205 153 *							[25]

T: Traces. * The greater amount with respect to the other compounds.

**Table 4:** Effect of butanol fraction of *P. peruviana* (BFPP) on fasting glucose, fasting insulin, and HOMA Index.

Treatment	Fasting Glucose mg/dL	Fasting Insulin (µUI/mL)	HOMA-IR
Normoglycemic	113 ± 5 ****	3.2 ± 0.3 ****	1 ± 0.1 ****
Vehicle	463 ± 18	27 ± 3.5	31 ± 4.1
Metformin 250 mg/kg	339 ± 29 ****	18 ± 3.9 ***	15 ± 2.5 ****
BFPP 50 mg/kg	335 ± 37 ****	11 ± 1.3 ****	9 ± 0.4 ****
BFPP 100 mg/kg	299 ± 18 ****	11 ± 1.4 ****	8 ± 1 ****

The data are expressed as mean ± SD. n = 6 animals per group. One-way ANOVA post-test Dunnet; **** *p* ≤ 0.0001 or *** *p* ≤ 0.001 compared with the vehicle group.
